# Iron deficiency anemia, stunted growth, and developmental delay due to avoidant/restrictive food intake disorder by restricted eating in autism spectrum disorder

**DOI:** 10.1186/s13030-020-00182-y

**Published:** 2020-04-10

**Authors:** Yoshitoki Yanagimoto, Yuko Ishizaki, Kazunari Kaneko

**Affiliations:** Department of Pediatrics, Kansai Medical University Medical Center, 10-15 Fumizonocho, Moriguchi, Osaka 570-8507 Japan

**Keywords:** Autism spectrum disorder, Avoidant/restrictive food intake disorder, Restricted eating, Iron deficiency anemia

## Background

Autism spectrum disorder (ASD) is characterized by impairments in social communication, restricted repetitive and stereotyped patterns of behavior, interests and activities by impairment of imagination, and hyper and hyposensitivity. Food refusal, restricted eating, and problems of eating habits and patterns are more observed in ASD than typical development [1]. Of the characteristics, restricted eating is attributed to restricted behavior and mouth hypersensitivity [2]. Hypersensitivity in patients with ASD often involves the intraoral sense of touch, particularly texture and consistency [3]. Although most cases achieve spontaneous remission [4], severe malnutrition leading to stunted growth can sometimes occur in children with ASD [5, 6].

Cases of children with ASD complicated by restricted eating resulting in severe malnutrition are rarely reported. Here, we report the case of a 2-year-old boy with ASD with severe iron deficiency anemia (IDA) and stunted growth caused by restricted eating.

## Case presentation

A 2-year-old boy presented with restricted eating, short stature, and failure to thrive. A local health center had diagnosed his condition as developmental delay and stunted growth. He was unable to eat any solid food and for the previous year had obtained his nutrients from breast milk and vegetable juice. He drank only specific brands of juice (only with his favorite tableware). He was taking no regular medication. Estimated daily energy intake based on dietary habits was approximately 650 kcal/day. His developmental history revealed language delay, obsessions, and repetitive behaviors. According to family history, his older brother had been diagnosed with ASD.

Physical examination revealed his height to be 74 cm (standard deviation [SD], − 3.9) and his weight to be 8.4 kg (SD, − 2.9). In addition, he looked pale and had tachycardia (132 bpm). Laboratory tests revealed severe microcytic hypochromic anemia (hemoglobin, 5.9 g/dL; mean corpuscular volume, 57.8 fL; mean corpuscular hemoglobin, 13.9 pg; serum iron, 14 μg/dL; ferritin, 7.5 ng/dL; TIBC, 447 μg/dL; and UIBC, 380 μg/dL). Two types of developmental assessment were performed. The Kyoto Scale of Psychological Development 2001 [7] revealed a developmental quotient (DQ) of 68. His Pervasive Developmental Disorders Autism Society Japan Rating Scale (PARS) score was 6 (above the ASD cut-off score). A developmental and behavioral pediatrics specialist diagnosed ASD using the Diagnostic and Statistical Manual of Mental Disorders (DSM-5) [8] diagnostic criteria and the PARS score. There was no evidence of suspected child abuse or maltreatment.

**Fig. 1:**
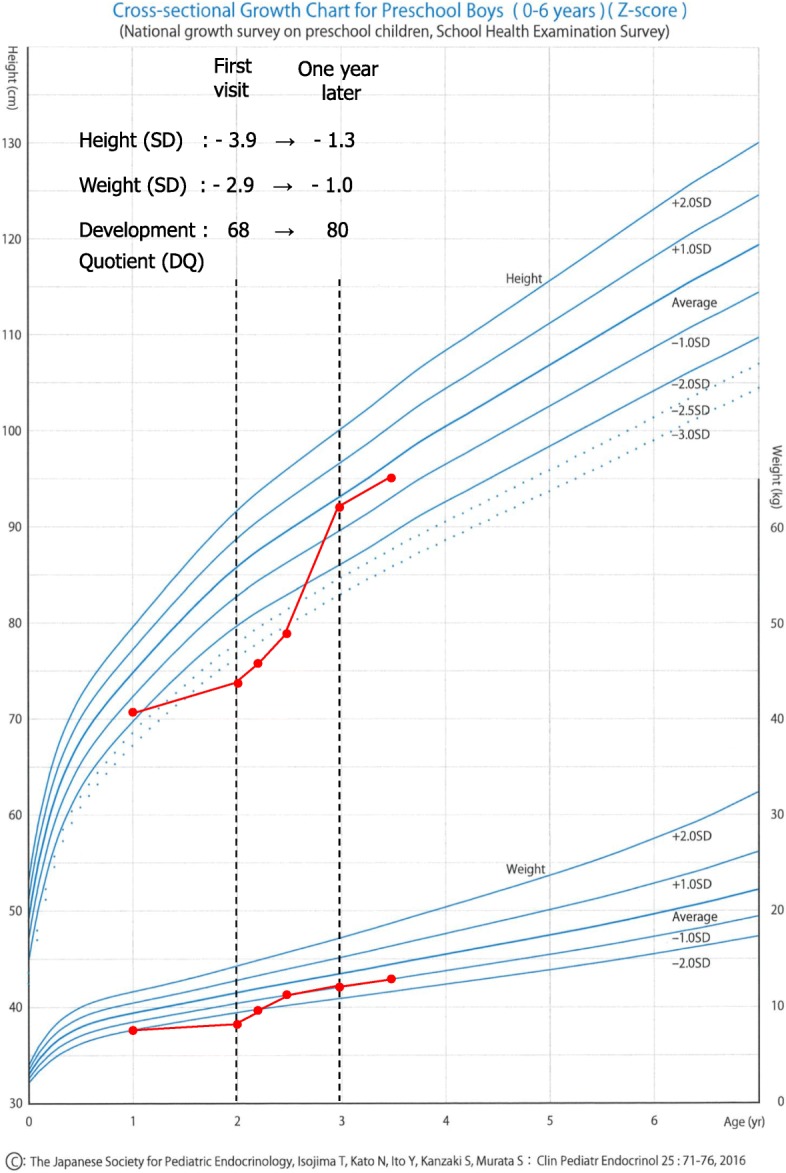
Clinical course of height, weight and deveropment

Based on these findings, the patient was diagnosed with IDA and malnutrition due to avoidant/restrictive food intake disorder (ARFID) (DSM-5) [8] related to ASD. We orally administered iron supplements (3 mg/kg/day) and enteral nutrients (600kcaL/day); fortunately, he took these by his favorite tableware without resistance. His anemia, nutritional condition, and clinical symptoms improved by treatment within 1 month. Hemoglobin, serum iron, and ferritin had also increased at that time to 10.5 g/dL, 168 μg/dL, and 32 ng/dL, respectively. Iron supplements were finished in 5 months and enteral nutrient were continued for 2 years. In parallel with nutritional replenishment, his height and weight improved to approximately − 1 SD, within the normal range, within 1 year (Fig. 1) [9]. In addition, he started eating a greater variety of foods, and his DQ and nutritional condition improved with nutritional replenishment. Hemoglobin, serum iron, and ferritin at that time had also increased to 12.6 g/dL, 108 μg/dL, and 21.2 ng/dL, respectively, all within the normal range.

## Discussion and conclusion

ARFID is an eating/feeding disturbance caused by apparent lack of interest in eating or food, avoidance based on the sensory characteristics of food, and concern about aversive consequences of eating [8]. Kristoffer reported that cases of ARFID caused by restricted eating or food refusal in ASD need forced nutrition therapy more frequently than those with typical development [10]. We successfully treated a 2-year-old boy with IDA and failure to thrive because of ARFID, which we considered to be caused by restrictive eating behavior and hypersensitivity associated with ASD. Hypersensitivity in patients with ASD often involves the intraoral sense of touch, particularly texture and consistency [3]. In this case, because he was able to drink specific liquids (breast milk and vegetable juice) and liquid forms of enteral nutrients we concluded that the consistency and taste of food and drink restricted his eating habits.

Previous studies have postulated various patterns of restrictive eating and deficiency (e.g., vitamin D deficiency [11]) and suggested that the incidence of iron deficiency is higher in ASD [12, 13]. Hence, restricted eating might lead not only to a lack of energy but also iron deficiency, resulting in IDA. However, there are no previous studies on this topic, except a few case reports and analyses of elemental deficiency in ASD, neither of which mention clinical problems.

He was able to take enteral nutrients without resistance. We think there are two reasons he was able to take enteral nutrients in spite of his severe food restriction. First, he took the nutrients in his favorite tableware. Second, he fortunately preferred the taste of the nutrients. In case of the patients with severe malnutrition, we should choose forced nutrition by nasogastric tube if sufficient nutrients cannot be taken orally..

It is of note that nutritional treatment improved not only his malnutrition and stunted growth but also his food selectivity and developmental delay, suggesting that malnutrition worsens developmental delay and food selectivity. We believe that malnutrition and anemia due to iron deficiency caused hypocirculation in the brain and digestive organs and promoted repetitive eating. As the IDA improved, physical growth and repetitive eating improved.

In conclusion, this case suggests that restricted eating is a risk factor for severe malnutrition, especially in ASD. We recommend that IDA and nutritional condition should be evaluated when an autistic child presents with restricted eating behavior and pallor.

## Data Availability

Not applicable.

## Abbreviations

ASD: Autism spectrum disorder
IDA: Iron deficiency anemia
ARFID: Avoidant/restrictive food intake disorder
